# Electrophysiological Characterization of Transport Across Outer‐Membrane Channels from Gram‐Negative Bacteria in Presence of Lipopolysaccharides

**DOI:** 10.1002/anie.201913618

**Published:** 2020-03-24

**Authors:** Jiajun Wang, Rémi Terrasse, Jayesh Arun Bafna, Lorraine Benier, Mathias Winterhalter

**Affiliations:** ^1^ Department of Life Sciences and Chemistry Jacobs University Bremen 28759 Bremen Germany; ^2^ Current address: School of Chemistry and Molecular Engineering East China University of Science and Technology 200237 Shanghai China; ^3^ School of Chemistry and Chemical Engineering Nanjing University 210023 Nanjing China

**Keywords:** biophysics, membrane channel, molecular transport, nanopore, permeability

## Abstract

Multi‐drug resistance in Gram‐negative bacteria is often associated with low permeability of the outer membrane. To investigate the role of membrane channels in the uptake of antibiotics, we present an approach using fusion of native outer membrane vesicles (OMVs) into a planar lipid bilayer, allowing characterization of membrane protein channels in their native environment. Two major membrane channels from *E. coli*, OmpF and OmpC, were overexpressed from the host and the corresponding OMVs were collected. Each OMV fusion surprisingly revealed only single or few channel activities. The asymmetry of the OMVs translates after fusion into the lipid membrane with the lipopolysaccharides (LPS) dominantly present at the side of OMV addition. Compared to the conventional reconstitution method, the channels fused from OMVs containing LPS have similar conductance but a much broader distribution and significantly lower permeation. We suggest using outer membrane vesicles for functional and structural studies of membrane channels in the native membrane.

## Graphical abstract

**Uptake of antibiotics**: To investigate the role of membrane channels in the uptake of antibiotics, outer membrane vesicles from *E. coli* were fused to a horizontal planar lipid bilayer. This method allows single channel recording in their native environment.

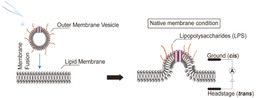

Multi‐drug resistant (MDR) bacterial pathogens, especially the “ESKAPE” family or the so‐called superbugs, are responsible for ca. two‐thirds of serious infection issues in clinic worldwide.^1^ Possible solutions involve improvement of antibiotic stewardship as well as new antibiotics.^2^, ^3^ As a response to the latter, the European Union supported a series of private‐public partnerships via the Innovative Medicine Initiative, building a platform called “New Drugs for Bad Bugs” (http://www.ND4BB.eu). One identified bottleneck in the development of new antibiotics against Gram‐negative bacteria is the low permeability across the cell wall and within the ND4BB platform; the “Translocation” project was devoted to investigating the low permeability issue.^4^

The envelope of Gram‐negative bacteria allows selective permeation of nutrients while protecting from toxic substances. Gram‐negative species contain a cellular inner membrane and an outer membrane with a peptidoglycan layer sandwiched in the middle. The outer membrane layer is structurally rigid due to the lipopolysaccharide (LPS) layer towards the environment. The LPS layer mainly protects the bacteria from certain chemical attack. Crystal structures showed that the LPS has a docking position at some of the outer membrane proteins (i.e., see for OmpF^5^). The conventional patch clamp technique is not suitable to study bacteria. One aspect is that the bacteria are too small, another aspect is that the LPS prevents the formation of a “giga‐seal” during the measurement. Moreover, artificial asymmetric membranes containing LPS to mimic the native environment are difficult to assemble and in particular, free standing bilayers are only possible for very short LPS.^6^, ^7^

One of the building blocks responsible for selectivity are channel‐forming outer‐membrane proteins facilitating the transport of hydrophilic molecules across the otherwise tight hydrophobic membrane.^8^, ^9^ In particular, major outer membrane channels such as OmpF and OmpC from *E. coli* are part of the pathway of antimicrobial influx.^10^, ^11^, ^12^, ^13^, ^14^ The high‐resolution crystal structure showed that OmpF is composed of three identical water‐filled monomers.^15^ Each monomer contains 16‐antiparallel β‐sheets spanning in the membrane domain. 8 long loops are connecting each pair of β‐sheets (L1–L8), among them, L3 folds almost into the halfway of the channel lumen forming the narrowest constriction region (CR). The L3 loop is mainly composed of negatively charged amino acid groups inducing slight cation selectivity in KCl under physiological conditions.^16^, ^17^, ^18^, ^19^ OmpC is a structurally related membrane protein with a smaller CR and expressed under extreme conditions by *E. coli* instead of OmpF.^20^

Recent advancements in proteomics provide quantitative numbers of the membrane proteins distribution in bacteria under various growth conditions.^21^ Previously, we characterized transport across an *E. coli* channel at a single protein level by reconstitution into an artificial lipid bilayer. To gain information on the mode of permeation, the ion current in absence and presence of small molecules was measured.^21^, ^22^ The contribution of the native environment such as LPS layer on the channel and on the permeability for small molecules across the outer membrane remains an open question.

Outer membrane vesicles (OMV) are spherical vesicles naturally secreted by Gram‐negative bacteria, involved in their survival under stress conditions and regulating microbial interactions within communities.^23^ Surprisingly, OMVs may fuse with lipid liposomes just by mixing.^24^ As they also contain outer membrane channels in their natural environment, we utilized them for channel characterization.^25^

Within the current urgent need for novel antibiotics, the key questions concerning the contribution of the so‐called porins to drug uptake are: why do some molecules permeate rapidly whereas others do not; which channels are involved; and what are the flux‐limiting interactions between antibiotics and channels? Recently established methods such as whole cell uptake assays and mass spectrometry provide an answer to the total penetration, which is the relevant parameter for the survival of a cell.^26^, ^27^ However, whole cell measurements reflect the sum of many possible pathways (e.g., porins, degradation enzymes, efflux pumps etc.). Optimization of the chemical structure concerning permeation remains difficult while relying only on an integrated set of data.

Here, we developed a direct approach to study porins in their native membrane using OMVs, with the goal to investigate to what extent the presence of LPS influences the flux of small molecules across porins. A further advantage is that such an OMV fusion approach is easy with respect to handling and may allow automatization.

Outer membrane vesicles were purified by differential centrifugation after overexpression of either OmpF or OmpC porin in *E. coli* BL21(DE3)omp8 bacteria. The purification process yielded samples containing the overexpressed porins as their major protein component (Figure 1 A) as previously shown mass spectrometry analysis.^25^, ^28^ In order to determine OmpF/C concentration in the samples, direct measurement of protein concentration was not possible since other proteins are also present. To circumvent this issue, we used densitometry on Coomassie‐stained SDS‐PAGE to determine the porins concentration (Figure 1 B). The results obtained show that our OMV suspensions contain fairly large amounts of OmpF (10 μm) or OmpC (8.5 μm). Dynamic light scattering (DLS) analysis of the OMV samples showed average sizes of 95 nm for OmpF‐containing OMV and 102 nm for OmpC‐containing OMV (Figure 1 C).

**Figure 1:**
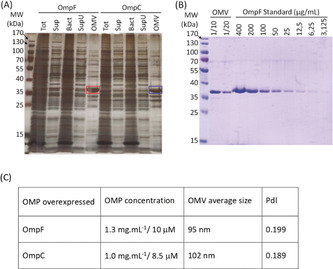
(A) Analysis of the protein content during the OMV purification process. Samples from total growth medium after 18 h incubation (Tot), supernatant after the initial low‐speed centrifugation and filtration (Sup), bacterial pellet (Bact), supernatant after high‐speed centrifugation (SupU) and resuspended OMV pellet (OMV, concentrated 500 times compared to the other samples) were separated by SDS‐PAGE and silver stained. Purifications of OMV from bacteria overexpressing OmpF and OmpC are shown. Red and blue rectangles highlight OmpF and OmpC proteins, respectively. (B) Measure of OmpF concentration in OMV. Diluted samples of OmpF‐containing OMV were analyzed by SDS‐PAGE along with a range of purified OmpF protein samples of known concentration. The SDS‐PAGE was Coomassie‐stained and the intensity of each OmpF band determined by densitometry (ImageJ). A standard curve was built from the purified OmpF samples and used to calculate the concentration of OmpF in the OMV samples. (C) Summary of OmpF‐ and OmpC‐containing OMV characterization. OMP concentration measured by SDS‐PAGE and OMV average size and polydispersity index (PdI) measured using a Malvern Zetasizer DLS instrument.

Inspired from early experiments with supported bilayer obtained via fusing small unilamellar vesicles, outer membrane vesicles were added directly towards the artificial lipid bilayer. In Figure 2 A–C we show schematic formation of OMVs. Figure 2 B shows fusion with a phospholipid membrane (see also Supporting Information) in 200 mm KCl, 20 mm MES and pH 6.0. Surprisingly, only few active channels were observed once fusion was obtained. In Figure 2 D we show the result of typical OmpF insertion. Likewise, in patch‐clamp, each vesicle fusion increases the conductance originating from an unknown number of porins (see Figure S1 in the Supporting Information). To distinguish single‐channel conductance, we applied higher transmembrane voltages to induce gating as typically observed for OmpF or OmpC in conventional symmetric bilayers.

**Figure 2:**
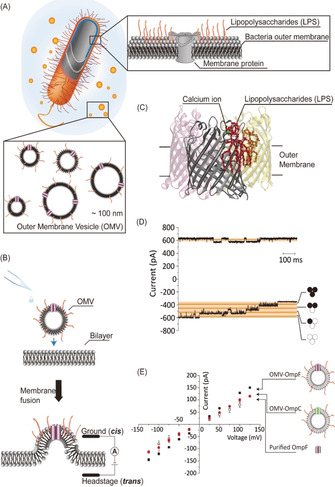
(A) Schematic outer membrane structure of Gram‐negative bacteria, composed of a phospholipids inner leaflet, lipopolysaccharides (LPS) outer leaflet as well as of membrane proteins. The bacilli secrete outer membrane vesicles (OMV) whose diameters are around 100 nm. It is expected that their composition is similar to the outer membrane.^23^ (B) OMVs are added directly to the planar lipid bilayer, forming BLM‐OMV. Fusion and protein activity is followed by applying external voltages. The ion current steps correspond to open porin insertion. (C) Crystal structure of LPS binding with OmpC homolog (5FVN) with each monomer in different color. Calcium (green) binding site suggests the involvement of divalent cation. Two LPS segments are plotted in red and orange. (D) Two typical ion current traces after OMV fusion containing likely three trimeric OmpF porins. Conclusions on single channel conductance are taken from the current steps caused under high applied voltage ±150 mV inducing gating. (E) I‐V response of OmpF and OmpC from OMV fusion. 200 mm KCl, 20 mm MES, pH 6.0 was used throughout. At least 3 individual experiments were performed.

The critical voltage necessary to observe channel gating was below −125 mV at a negative voltage or above +150 mV at a positive voltage, comparable to previous observations with purified porin reconstituted into an artificial bilayer. At a transmembrane voltage of −150 mV, the change of the average current baseline due to OmpF monomeric gating is 39±5 pA corresponding to 1.1±0.1 nS for a trimeric OmpF (see Figure S3). This observed conductance value is similar to purified OmpF reconstitution (0.8 nS at 200 mm KCl, pH 6.0).^19^, ^22^, ^29^, ^30^ Note that the statistical distribution using OMVs was significantly broader compared to single channel reconstitution.

To investigate whether this is a general feature of OMV reconstitution and not specific to OmpF, we then overexpressed OmpC, an OmpF homolog, in the same strain and fused the secreted OMVs to a planar lipid bilayer. The induced channel activity confirmed the trend for OmpF. The typical ion current versus applied voltage is plotted in Figure 2 E and compared with purified OmpF. Following the traditional purification–reconstitution protocol at 200 mm KCl and pH 6.0, we obtain for OmpF 800 pS and OmpC 600 pS, while the fusion of OMV showed conductance of OmpF 1100±100 pS and for OmpC 840±45 pS.^19^ Typically only 1–3 active channels were observed per OMV fusion events (number of experiments: *n*>20).

To elucidate possible stabilization effects of LPS in presence of divalent ions, we added 5 mm EDTA and incubated the vesicles at 4 °C overnight. Within the experimental error, we did not see a significant change (see Figure S3).

In a second series of measurements, we tested four otherwise well studied antibiotics to elucidate the effect of the LPS barrier.^30^ We first fused OMV containing OmpF to the lipid membrane and added Enrofloxacin (250 μm) to the same side as OMV addition. In agreement with previous single channel recording, interaction spikes were observed at a negative transmembrane voltage (Figure 3 A,B). A statistical analysis of the events provides the event rate (*k*_on_=12,000 s^−1^ m ^−1^) (Figure 4 A) and dissociation rate (*k*_off_=270 s^−1^) of Enrofloxacin interaction with the channel at −50 mV (Figure 5 A). Note that there is no interaction observed when a positive voltage is applied. Higher event rates are found using purified OmpF. Statistical analysis revealed *k*_on_ *=*72,000 s^−1^ m ^−1^ and *k*_off_=500 s^−1^ in agreement with earlier results.^7^, ^11^, ^30^ Such asymmetry in the measured kinetics hint towards a barrier presented by the LPS for the transport of substrate molecules across the outer membrane of Gram‐negative bacteria. It also demonstrates that the LPS is facing towards the addition of substrate side. The event rate of Enrofloxacin drops by almost 6 times in the presence of LPS and slows down the translocation of the substrate molecule by almost 50 %, see Figure 3 (A and B) for current trace comparison and Figure 4 A for event rate and Figure 5 A for dwell time comparison. To enhance the signal to noise ratio in electrophysiology for single channel measurements, we used a higher salt concentration (1 m KCl). We further studied other antibiotics like Norfloxacin, Ciprofloxacin and Kanamycin sulfate through OMV‐OmpF and compared it with the OmpF‐WT. The chemical structures of the analytes are illustrated in Figure S6. In Figure 3 C,D we show Kanamycin sulfate permeation for both batches of preparation, almost one fold reduction in event rate compared to OmpF‐WT was observed (Figure 4 B). The dwell time comparison of Kanamycin sulfate through both OmpF‐WT and OMV‐OmpF follows a similar trend and no voltage dependency was observed in both cases, indicating negligible permeability across OmpF (see Figure 5 B). With Norfloxacin, an event rate with OMV‐OmpF with 50 % enhanced dwell time compared to OmpF‐WT (Figure 4 C) was observed (Figure 5 C), corresponding current traces (see Figure S4 A). In case of Ciprofloxacin, no significant change in the event rate was observed for both OMV‐OmpF and OmpF‐WT (see Figure 4 D), but dwell time analysis showed nonlinear behaviour (see Figures 5 D and S5), which hints to the absence of translocation in case of OMV‐OmpF (for current trace see Figure S4 B). To draw a quantitative conclusion on effective translocation from event rates requires caution as many spikes in the ion current trace rather correspond to bounce back events. Hence, we need to inspect carefully the dwell time statistics to extract information on actual translocation (note that increasing dwell times with increasing transmembrane potential suggest true translocation).

**Figure 3:**
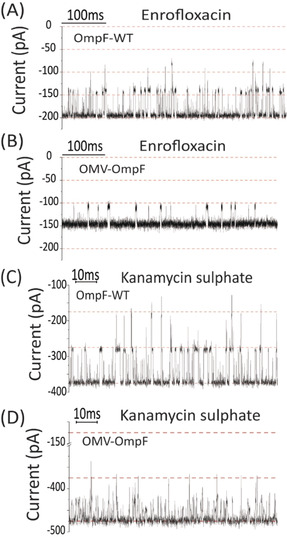
Comparison of ion traces after single protein reconstitution vs. channel reconstitution by fusion of OMVs. (A) OmpF‐WT and (B) OmpF‐OMV both in 250 μm Enrofloxacin (200 mm KCl + 20 mm MES at pH 6) applied voltage −100 mV. (C) OmpF‐WT and (D) OmpF‐OMV. Ion current trace in presence of 10 μm Kanamycin sulfate, (1 m KCl + 20 mm MES at pH 6) applied voltage −100 mV. (The chemical structures are given in the Supporting Information).

**Figure 4:**
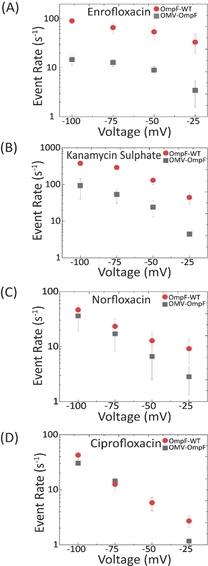
Event rate comparison of single protein reconstitution vs. channel reconstitution by fusion with OMVs. (A) Event rates of Enrofloxacin (250 μm) in 200 mm KCl + 20 mm MES. (B) Event rates of Kanamycin sulfate (10 μm) in 1 m KCl + 20 mm MES. (C) Event rate of Norfloxacin (250 μm) and (D) Ciprofloxacin (250 μm) both in 1 m KCl + 20 mm MES. All measurements were carried out at pH 6.

**Figure 5:**
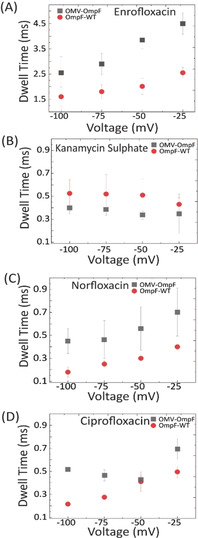
Dwell time comparison of single protein reconstitution vs. channel reconstitution by fusion with OMVs. (A) Dwell time of Enrofloxacin (250 μm) in 200 mm KCl + 20 mm MES. (B) Dwell time of Kanamycin sulfate (10 μm) in 1 m KCl + 20 mm MES. (C) Dwell time of Norfloxacin (250 μm) and (D) Ciprofloxacin (250 μm) both in 1 m KCl + 20 mm MES. All measurements were carried out at pH 6.

In a further set of experiments, we followed another approach by fusing OMVs with giant unilamellar vesicles (GUVs) leading to giant vesicles with a typical final diameter in the range of 10 to 20 μm, large enough to be used in a patch‐clamp setup. Under the microscope, the vesicles appeared spherical and were subsequently patched in the Port‐a‐Patch (Nanion Technologies GmbH, Germany). Both OmpF and OmpC channel activities could be obtained from their gating behaviour at high voltages (Figure S1) and were comparable with the OMV fusion (Figures S2).

According to previous studies using the conventional purified protein reconstitution approach,^31^, ^32^ the extracellular side of the channel from OMV fusion is the same as the sample addition side, which is identical to the mechanism of the purified protein reconstitution, as illustrated in Figure 2 C. Since the channel orientation from OMV fusion is the same as with the conventional reconstitution method, we conclude that both the LPS and channel extracellular side face towards the *cis* side, which is the OMV addition side. This orientation will help further understanding of the electrostatic interaction between LPS and the antibiotic molecules when treated under different buffer conditions.

Based on our results described above, we suggest a direct approach to study membrane proteins in their native environment. Fusion of OMV with planar lipid bilayer allows single or few porin insertion. The reason for such an unexpected low porin number might be an intrinsic selection for single channel reconstitution. Other conditions resulted in 2D crystals, which in all likeliness are not readily able to fuse with a lipid bilayer.^**[**25**]**^ Single‐channel current traces resulted in somewhat lower conductance and with a broad standard deviation compared to purified and reconstituted single membrane protein. We compared single channel translocation data of OmpF‐OMV with purified OmpF‐WT and identified a barrier effect: lower event rates and slower translocation times. We expect that this approach is also valid for many other porins to study not only the permeation of small drug molecules but the single entity electrochemical behavior.

## Conflict of interest

The authors declare no conflict of interest.

## Supporting information

As a service to our authors and readers, this journal provides supporting information supplied by the authors. Such materials are peer reviewed and may be re‐organized for online delivery, but are not copy‐edited or typeset. Technical support issues arising from supporting information (other than missing files) should be addressed to the authors.

Supplementary
