# Supplementary material for: Electrophysiological Characterization of Transport Across Outer‐Membrane Channels from Gram‐Negative Bacteria in Presence of Lipopolysaccharides
Source: Angew Chem Int Ed Engl. 2020 Mar 24;59(22):8517–21. doi: 10.1002/anie.201913618 (PMC7317717; doi:10.1002/anie.201913618)
Supplement: Supplementary file 1 — Supplementary [file ANIE-59-8517-s001.pdf]

Angewandte Chemie
Eine Zeitschrift der Gesellschaft Deutscher Chemiker

Supporting Information

Electrophysiological Characterization of Transport Across Outer-Membrane Channels from Gram-Negative Bacteria in Presence of Lipopolysaccharides

*Jiajun Wang, Rémi Terrasse, Jayesh Arun Bafna, Lorraine Benier, and Mathias Winterhalter**

anie_201913618_sm_miscellaneous_information.pdf

Supporting information

Experimental Section

Chemicals

For electrophysiology experiments, 1,2-diphytanoly-snglycero-3-phosphocholine (DPhPC) from Avanti polar lipids, Inc. (Alabaster, AL) was used. Chloroform, lysogeny broth (LB), ampicillin, isopropyl β-D-1-thiogalactopyranoside (IPTG), 2-(N-Morpholino) ethanesulfonic acid hydrate (MES hydrate), sodium chloride (NaCl), and potassium hydroxide (KOH) was obtained from Roth (Carl Roth GmbH, Germany). Potassium chloride (KCl), potassium acetate, 4-(2-hydroxyethyl)-1-piperazineethanesulfonic acid (HEPES), and Norfloxacin, Ciprofloxacin, Enrofloxacin and Kanamycin sulphate were obtained from Sigma (Sigma-Aldrich, Germany). 1% n-octylpolyoxyethylene (Octyl-POE, Bachem) with 20 mM potassium phosphate, pH 7.4 was used for the dilution of the purified protein for reconstituting single proteins in an artificial phospholipid bilayer.

Outer membrane vesicles preparation

Production of OMV was performed in E. coli BL21(DE3)omp8 (ompF::Tn5 ΔompC Δ ompA ΔlamB) in order to reduce background noise from endogenous porins.33 OMV containing OmpF or OmpC were obtained from bacteria transformed with pGOmpF or pGOmpC,34 respectively. Two 500 mL flasks containing 250 mL of LB + 100 μg/mL ampicillin were inoculated with 2.5 mL of an overnight culture in the same medium and grown 3h at 37°C with 200 rpm shacking. OmpF or OmpC expression was induced by adding IPTG at a final concentration of 400 μM. The cultures were then grown for an additional 18h before purification of OMV.

OMV purification was adapted from Chutkan *et al.* [1] The bacterial cultures were centrifuged 45 min at 3,220 g to pellet bacteria. The supernatants obtained were then filtered through a 0.22 μm PES bottle top vacuum filter (Corning) to remove residual bacteria. OMV were then pelleted by centrifugation at 40,000 g for 2h (18,600 rpm in 45 Ti Fixed-Angle rotor). The supernatants were carefully decanted and OMV pellets re-suspended in 500 μL of 10 mM HEPES, 150 mM NaCl, pH 7 buffer. The OMV suspensions obtained were filter-sterilized using

0.22 μm PVDF centrifugal filters (Ultrafree-MC, Millipore), aliquoted and stored at -80°C. Purity and homogeneity of the OMV was confirmed by SDS-PAGE and DLS (not shown).

Giant unilamellar vesicles (GUV) preparation and OMV reconstitution

For the electrophysiological characterization we tried two protocols, one fusing OMV´s to giant lipid vesicles (See SI) and one direct fusion to a pre-existing membrane. GUVs whose size are around 20 µm formed using electroformation was used as bilayer domain for the fusion of OMVs. Specifically, GUVs formed in the presence of 1 M Sorbitol was collected from the ITO slides and checked its uniformity under optical microscope. OMV samples was incubated with the GUV solution at a ratio 1:25 (v:v) overnight at 4° C . Then the incubated sample were used for planar lipid bilayer experiment using Port-a-patch (Nanion Technologies GmbH, Germany). A borosilicate glass chip with thickness 125 µm and 1 µm diameter hole could act as the patch clamp tip to catch a GUV under a negative pressure. Direct patch with OMV is not possible since the OMV size (100 nm diameter) is too small for one patch clamp experiment.

Artificial lipid bilayer formation and OMV fusion

Planar artificial lipid bilayer was formed using 5 mg/ml DPhPC/octane (w/v) on Orbit mini (Nanion Technologies GmbH, Germany). 0.5 µL vesicle samples (i.e. OMVs) were added directly to the measuring chamber from the ground (cis) side. Under the application of membrane voltage (150mV), we see a sudden jump in the bilayer conductance and that corresponds to successful fusion of OMV's to the artificial lipid bilayer. . All the electrophysiology measurement has been monitored and recorded using Element Data Recorder software (Element s.r.l., Italy) and further analyzed using Clampfit 10.7 software (Axon Instrument Ltd., US). In the first series of measurement we mainly used 200 mM KCl, 20 mM MES at pH 6.0, to increase the resolution we used higher salt solution (1 M KCl, 20mM MES at pH 6).

Reconstitution of OMV into GUVs

Giant uni-lamellar vesicles (GUVs) were prepared using DPhPC (Avanti Polar Inc.) following electro-formation protocol previously introduced.[2] Briefly, 10 μL DPhPC (5 mg/ml) was dissolved in chloroform and deposited on the conductive part of the ITO slides. After complete dehydrated, an O-ring was placed around the lipid residue filled with 1 M sorbitol. The vesicle was prepared under Vesicle prep pro (Nanion Technologies GmbH, Germany).
To fuse the OMVs into the GUVs, 10 μL OMV containing OmpF or OmpC sample solution was incubated with 500 μL GUV solution overnight at 4°C following the previous purified protein protocol.[3] Then the reconstituted GUV solution was used for patch clamp system.

The Port-a-patch (Nanion Technologies GmbH, Germany) was used to characterize the GUV fused with OMVs containing membrane protein channels. A borosilicate glass chip with an aperture of 1 μm is fabricated, a negative pressure is applied to attract the sample vesicles onto the aperture for electrophysiological characterization.
Briefly, 5 μL buffer solution (200 mM KCl, 20 mM MES, pH 6.0) was added on both sides of a borosilicate chip. Another 5 μL GUV sample solution was added to the cis side of the system, immediately a -25 mbar was applied to get a good giga-seal resistance. After a stable seal resistance was achieved, the pressure is slightly increased until the vesicle ruptured on the borosilicate glass chip.[4] The current signal was recorded by Axon 200B (Axon Instrument Inc.).

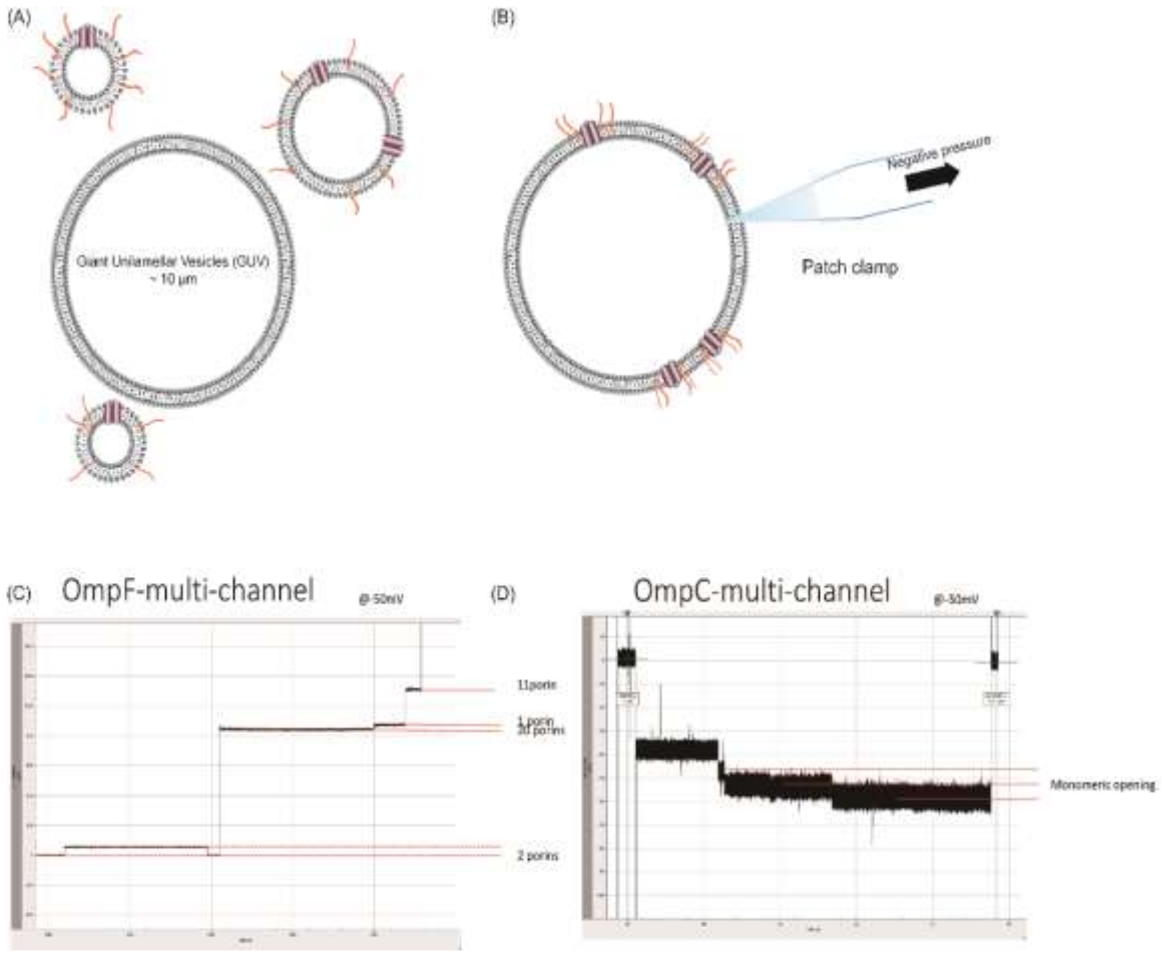

Figure S 1: (A) OMV fusion with Giant uni-lamellar vesicle (GUV). (B) Such bacteria outer membrane mimic at mammalian cell size can be studied using conventional patch clamp technique. (C) Multichannel activity of OmpF from OMV fusion is recorded. (D) Multichannel activity of OmpC from OMV fusion is recorded. 200 mM KCl, 2 mM MES, pH 6.0 as measuring condition. At least 3 individual experiments have been performed.

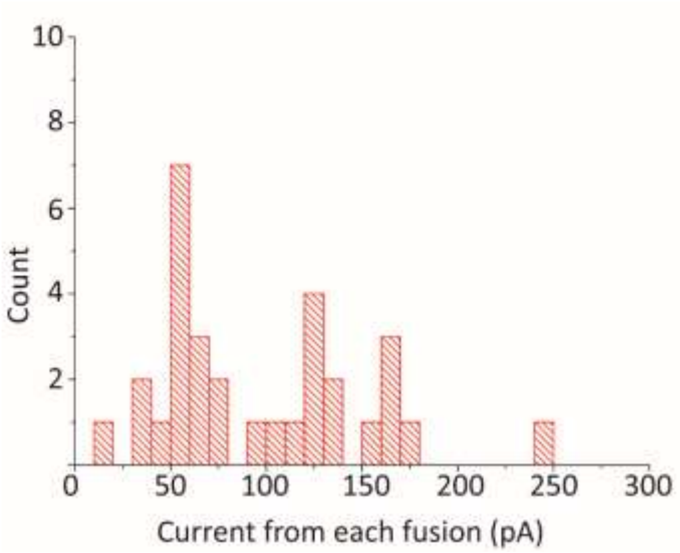

Figure S2: Histogram of current from each OmpF containing OMV insertion. 200 mM KCl, 20 mM MES, pH 6.0 was used as measuring condition. The histogram shows more than 20 times fusion from the proteo-OMVs, 3 main peaks are obtained accumulated at 53 pA, 120 pA, 160 pA respectively. From the histogram analysis, the OMV fusion providing 1 protein activity shows the highest possibility, following twice the amperometric response then triple, which demonstrates that only few protein activity could be obtained from OMV fusion to the planar lipid bilayer.

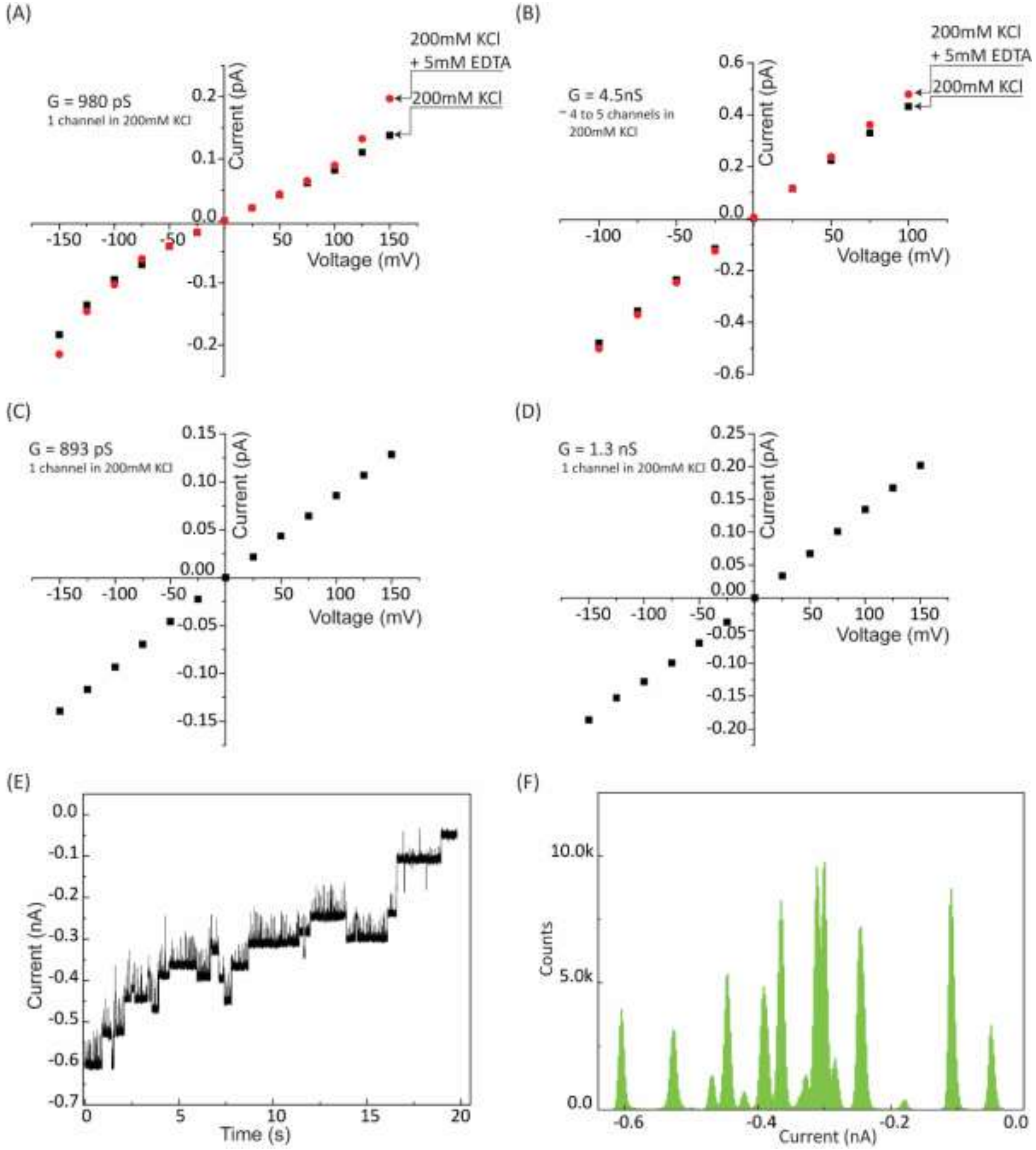

Figure S3: Effect of EDTA on OMVs. (A,B,C and D) The I-V of planar bilayer after OMV fusion (OmpF) (200 mM KCl, 20 mM MES, pH 6.0 with and without 5mM EDTA). (E) is the stepwise gating of OmpF at -150mV (multiple activities of OmpF in OMV fusion). (F) is the corresponding histogram of current trace shown in (E).

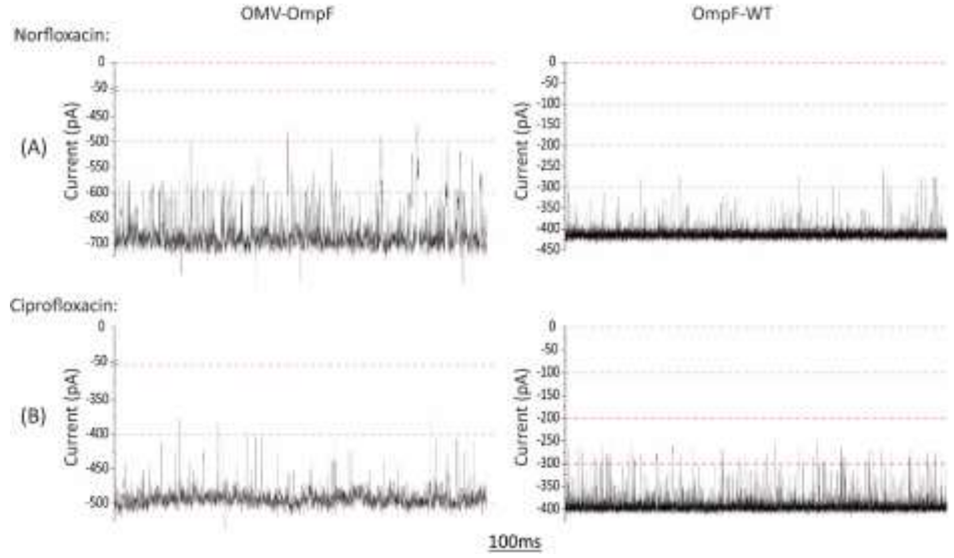

Figure S4: (A) is the current trace for OMV-OmpF and OmpF-WT in presence of Norfloxacin (0.25 mM) on cis side of the membrane. (B) is the corresponding current trace for Ciprofloxacin.

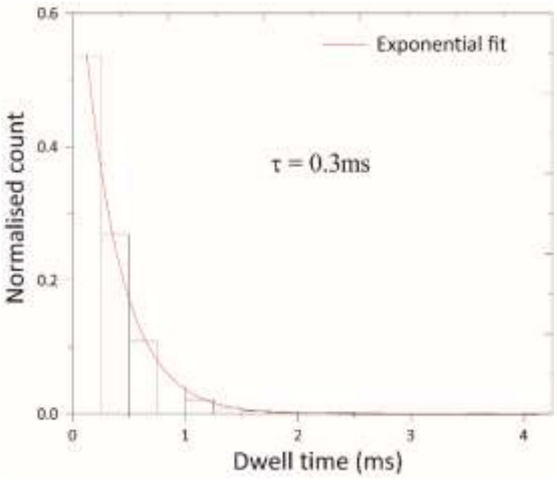

Figure S5. Typical dwell time histogram with exponential fit (red line) for OMV-OmpF with 250µM Norfloxacin at -100mV applied potential. [5,6]

(A) 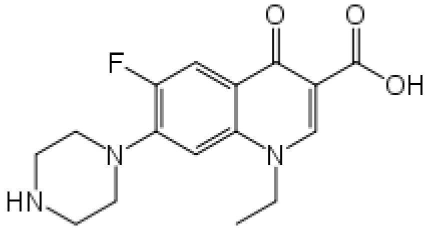

(B) 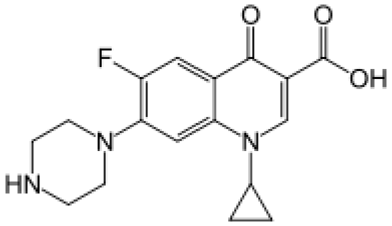

(C) 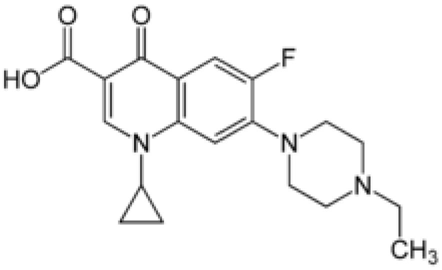

(D) 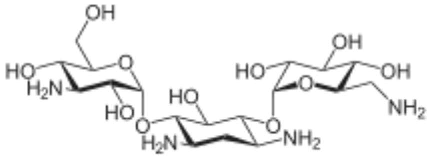

Figure S6. Chemical structure of (A) Norfloxacin (B) Ciprofloxacin (C) Enrofloxacin and (D) Kanamycin

Reference

1. Chutkan, H., et al., Methods Molecular Biology, 2013, 966, 259-72.
2. Wang, J., L. Benier, and M. Winterhalter, *Quantifying Permeation of Small Charged Molecules across Channels: Electrophysiology in Small Volumes.* ACS Omega, 2018. **3**, 17481-17486.
3. Weichbrodt, C., *et al. Antibiotic translocation through porins studied in planar lipid bilayers using parallel platforms.* Analyst, 2015. **140**: p. 4874-81
4. Mahendran, K.R., *et al.. Permeation of antibiotics through Escherichia coli OmpF and OmpC porins: screening for influx on a single-molecule level.* Journal of biomolecular Screening, 2010, **15**, 302-307.
5. Colquhoun D, Hawkes AG The Principles of the stochastic interpretation of ion-channel mechanisms. In Bert Sakmann and Erwin Neher. Single-channel Recording pp 397–482. Boston, MA: Springer (1995).
6. Mahendran, K.R., *et al. Antibiotic translocation through membrane channels: temperature-dependent ion current fluctuation for catching the fast events.* European Biophysics J. 2009, **38**, 1141-1145
